# The outcomes of postgraduate palliative care education and training: assessment and comparison of nurses and physicians

**DOI:** 10.1186/s12904-023-01217-1

**Published:** 2023-07-13

**Authors:** Kevin Marciniak, Alexandra Scherg, Piret Paal, Stephen Mason, Frank Elsner

**Affiliations:** 1 Department of Palliative Medicine, Medical Faculty RWTH Aachen University, Aachen, Germany; 2 Department of Critical Care and Emergency Medicine, Klinikum Bremen Mitte, Bremen, Germany; 3 Institute of Palliative Care, Paracelsus Medical University in Salzburg, Salzburg, Austria; 4 Palliative Care Unit, School of Medicine, Institute of Life Course & Medical Sciences, University of Liverpool, Liverpool, England

**Keywords:** Evaluation, Palliative care, Symptom control, Learning gain, Interprofessional education, Germany

## Abstract

**Background:**

Within Germany, there is a heterogeneous range of training and continuing education in palliative care for different professional groups. The German Society for Palliative Medicine (DGP), together with the German Hospice and Palliative Care Association (DHPV), have defined quality requirements for postgraduate training in palliative care. These requirements include the evaluation of course structures and the assessment of outcomes.

**Aim:**

To assess the ‘learning gains’ in palliative care nurses and physicians undertaking continuing education programmes, and evaluate the structures and processes. To identify if/how the continuing education programmes could be improved.

**Material and methods:**

The development of Nurses’ and Physicians’ learning was determined using a retrospective self-assessment procedure. The evaluation was based on learning objectives developed in the DGP Education Working Group, using a six-point Likert scale for each item, and space for ‘free-text’ comments. Assessments were conducted after training.

**Results:**

Five hundred twenty nine self-assessments were recorded (456 nurses / 73 physicians). An increase in learning is demonstrated in all areas (knowledge, skills, social and self-competence) for each profession. The greatest gain was in symptom control. However, there were significant differences in the extent of learning gains between nurses and physicians.

**Conclusion:**

Analysis suggests current training results in improvements, but personal competences progress less than knowledge and skills. One way to improve this would be to introduce more interprofessional continuing education elements. Evaluation, as a basis for improving training concepts, is essential for continual development.

**Supplementary Information:**

The online version contains supplementary material available at 10.1186/s12904-023-01217-1.

## Background

The World Health Organisation’s (WHO) definition of Palliative Care [1] indicates that for people with serious illness, the best possible care is achieved through interdisciplinary approaches. The WHO definition does not include the concept of education, even though educating the general public and health professionals about palliative care is crucial [2]. However, the WHO Framework of Interprofessional Education (IPE) encourages two or more professions to learn with, from and about each other in order to improve collaboration and quality of care [3].

Palliative care is facing new challenges that education and training programmes must address, including: the need for early integration, demographic changes, treatment of patients with non-malignant disease, complex symptom patterns and multiple co-morbidities [4, 5]. Since 2013 palliative medicine has been a compulsory subject for German medical schools [6]. In nursing schools, palliative care (20 h) became a compulsory subject in 2019 [7]. Therefore, many people currently working in health care may have received little or no training in palliative care. In Germany, medical students are trained at universities, and nurses at universities of applied sciences. Consequently, opportunities for interactive learning with different professional groups are limited. As healthcare professionals are confronted with death and dying in all healthcare settings, the lack of competencies in the existing workforce in palliative care is a problem that may be addressed by enabling access to interprofessional postgraduate education.

### German palliative care structures

Founded in 1994, the German Society for Palliative Medicine (DGP) [8] is the first medical-scientific professional society in Germany. Members of the DGP include physicians and members of other professional groups (as of 31 December 2019, a total of 6151 DGP members, including 3334 physicians, 1866 nurses, 919 Allied Health Care Professionals and 32 supporting members [9]). The German Society for Palliative Medicine (DGP) enables certification of postgraduate training and education according to defined specific framework conditions. In addition, the DGP together with the German Hospice and Palliative Care Association (DHPV) has defined quality requirements for postgraduate training in palliative medicine and palliative care, including evaluation and outcome assessment [10].

### Quality requirements, competency fields and palliative care core competencies

In order to improve the quality of postgraduate training, the DGP's Education Working Group has defined learning objectives for interprofessional education in the *Kompetenzbasierte berufsgruppenunabhängige Matrix zur Erstellung von Curricula für die Weiterbildung curricularer Bildungsinhalte in Palliative Care / Palliativmedizin* (KoMPaC) [11]. The learning objectives in KoMPaC are based on the ten interdisciplinary core competencies of the European Association of Palliative Care [12], as well as on the competence fields of the German Qualifications Framework (DQR) for lifelong learning [13]. The DQR framework was developed by the Federal Ministry of Education and Research, and the Standing Conference of the Ministers of Education and Cultural Affairs of the Laender in the Federal Republic of Germany, to make the German education system more transparent and enable the classification of various qualifications in a national and international context.

According to the DQR, there are four competency fields (Knowledge, Skills, Social, and Self-competence) which are grouped into two competence categories (Professional and Personal competencies). Each competency field should assess the participants in relation to the demanded core competence as follows (see Table 1):

- Knowledge – Breadth and depth of knowledge
- Skills –Systemic and instrumental skills, amenable to evaluation
- Social competence – Leadership, team-working and communication
- Self-competence – Autonomy/responsibility, reflexivity and learning competence

**Table 1:** Exemplary representation of the learning objective and item development

Competence field according to the DQR		Core competence 2: “Enhance physical comfort throughout the patients´ disease trajectories.”
Professional competence	Knowledge	Item 2: “I can explain the Total Pain concept in detail.”
	Skills	Item 12: “I develop individual strategies to actively support the patients´ wellbeing and quality of life to maintain
Personal competence	Social competence	Item 22: “I always perceive and acknowledge the patients´ individual symptom perception and suffering experience.”
	Self-competence	Item 32: “I essentially respect my own and others’ limits.”

The KoMPaC applies the core competencies of palliative care to the competence fields of the DQR quality framework. This enables the development of structured learning objectives for the individual competence fields that will achieve appropriate and needs-based care for the seriously ill and dying, according to the requirements of WHO [2, 11, 12] (see Table 2).

**Table 2:** Item-development via connecting competency field of the DQR and the palliative care core competencies

**Competency field** according to the German Qualifications Framework for Lifelong Learning		** + **	**10 core competencies of palliative care**
Professional competencies	Knowledge		1. Apply the core components of palliative care in the setting in which patients and families find themselves 2. Improve the physical wellbeing of patients throughout the course of illness 3. Meet the psychological needs of patients 4. Meet the social needs of patients 5. Meet the spiritual needs of the patient 6. Respond to the needs of family carers in relation to short, medium and long-term patient care goals 7. Respond to the challenges of clinical and ethical decision making in palliative care 8. Practice comprehensive care coordination and interdisciplinary teamwork in all settings where palliative care is provided 9. Develop interpersonal and communication skills appropriate to palliative care 10. Practice self-awareness and engage in professional development
	Skills		
Personal competencies	Social competence		
	Self-competence		

Due to growing demand for care, a heterogeneous range of continuing education courses in palliative care are offered by different providers in Germany. Some nursing courses are certificated by the German Society for Palliative Medicine, some are not, and there are rarely legal demands concerning the content and structure of the courses [14, 15]. The learning objectives in physicians postgraduate qualification is determined, and examined, by the German Medical Association. After graduation physicians can obtain a specialisation in palliative medicine. A career path for nurses, such as advanced practitioner in palliative care, is not yet established.

### Aim/objective

This study aims to assess the ‘learning gains’ of nurses and physicians in palliative care continuing education programmes and evaluate the structures and processes of the education provided. Such data will help to understand if and how the continuing education programmes could be improved.

## Material and methods

### Assessment tool(s)

An assessment tool employing a 6-point Likert scale (where 1 = Best, and 6 = Worst) across 40 items made up of a combination of the KoMPaC and the DQR framework, was developed by the authors for this study in 2018. The assessment was developed as a retrospective tool using a ‘post-then approach’ and it provides the opportunity to calculate the improvement in percentage for illustration; via the application of a technique called Comparative Self-Assessment-Gain (CSA-Gain). In this study, we were able to use data from 529 completed assessments. Besides the structured evaluation via CSA-Gain, we offered the participants the ability to provide their thoughts via a free-text response field. For qualitative evaluation of the free-text answers, we used coding and content analysis, following the process outlined by Saldana [16].

Every item in the assessment addresses a KoMPaC field or learning target with a 6-point Likert scale where the participants should evaluate their knowledge, skills, social and self-competence before and after completing the continuing education programme (pre and post). We built the scale analogical to the German scholar-grade system (1 = the best grade, anchored left and 6 = the worst grade, anchored right)—see Supplementary information: Additional file 1. Overall, we grouped the items according to the competency fields.

- Items 1-10 = Knowledge
- Items 11-20 = Skills
- Items 21-30 = Social competency
- Items 31-40 = Self competency

### Procedure

Two forms were developed for the evaluation of palliative care training in Germany, which were intended to map all dimensions of the evaluation (structures, processes, teaching and outcomes) and weight them accordingly [17–19]. One form covered the dimensions of structures, processes and teaching, and the second form the outcome. We split the forms to avoid the influence of the experience during the course upon the targeted outcome. In this paper, only the specific outcome of the extent of learning development is described. The form used to access these data was purposively created for this study and is accessible in the supplementary files: a translated version was used by Paal et al. for a similar study in the Ukraine [20].

We randomly asked providers of DGP-certified continuing education programmes in palliative care across Germany to participate in this study. The courses we examined were attended either by physicians or nurses, there was no joint participation in the sense of Interprofessional continuing education. The assessment tool(s) were distributed by the course instructors to all participants attending the courses. We did not record the response rate in detail, and using existing participation rates we can reliably estimate that the response rate is approximately 80% per course.

The assessment tool contained participant information on the aim of our study, identifying that participation was voluntary and that all data would be treated anonymously. Participants were then asked to declare which occupational group they belong to. Apart from this, no other personal details (age, gender, experience, etc.) were requested to ensure anonymity. In terms of the participants´ experience, all physicians were licensed and all nurses were qualified via a state examination. However, nurses with this degree cannot be compared with holders of the Clinical Nurse Specialist or Advanced Practice Nurse designations [21]. At the end of a course, participants rated their level of knowledge before and after the training in relation to the specific learning objectives. After the 40 assessment items, participants had the opportunity to provide a free-text answer/evaluation. Participants were asked to address how their assessments would have been before the education, and their assessments following the education.

### Learning gain calculation via comparative self-assessment (CSA-Gain)

We compared the improvements in learning for nurses and physicians, and compared between groups for differences. This analysis aimed to identify areas of specialised education that work well and areas that may need revision.

The outcome assessment was executed employing a comparative retrospective (post-test) self-assessment after completing the continuing education programme [22, 23]. Implementing only one evaluation point in time makes the procedure more user-friendly, so a higher participation rate can be expected. Furthermore, a work by Schiekirka showed that a pre/post survey at a single point in time (after the training) is not inferior to traditional pre-post surveys, and leads to equal outcomes [24, 25]. Learning gains for each learning objective were calculated using the following formula, accounting for prior learning [25, 26]:

$$\mathrm{CSA\;Gain\;}[\mathrm{\%}] = \frac{\upmu\;pre-\upmu\;post}{\upmu\;pre-1}\mathrm{ x }100$$

Here the participants' self-assessed level of knowledge before (μ pre) and after (μ post) the course are used. The difference between the mean pre-ratings and the mean post-ratings is divided by the mean pre-rating minus 1 and then multiplied by 100 to get a percentage. To take different levels of pre-course knowledge into account the formula uses a division by the mean pre-ratings [25, 26]. The formula shown above works with a scale from 1 to 6 (1 = the best grade, 6 = the worst grade).

One core element of the formula is to calculate learning gain independent of the amount of prior knowledge. The following example in Table 3 shows how this adjustment can be achieved.

**Table 3:** Example CSA Gain with 3 levels of prior knowledge (low, mid and high)

Pre-Rating	Post-Rating	Absolute difference	Adjusted pre-rating	CSA Gain
5	3	2	4	2/4 = 50%
4	2.5	1.5	3	1.5/3 = 50%
2	1.5	0.5	1	0.5/1 = 50%

The example shows that a certain Comparative Self-Assessment Gain (e.g. 50%) can be reached via variable absolute changes from pre to post-ratings depending on prior knowledge [27]. Higher prior knowledge levels need lower absolute differences to reach a 50% gain, than low prior knowledge levels. A post-rating of 1 will lead to a CSA or learning gain of 100% irrespective of pre-ratings.

### Qualitative analysis of free text

The evaluation included a free-text field that was frequently used to address dimensions not explicitly assessed (structures, processes, teaching). The free-text answers were analysed with the help of evaluation coding [16]. Table 4 illustrates how we developed the coding. For analysis, we used first cycle methods—magnitude and descriptive coding. The magnitude coding shows if the evaluation consists of positive or negative feedback. Via descriptive coding, all free-text evaluations were grouped to the addressed topic. Furthermore, the In Vivo coding was applied to extract short phrases as recommendations or summaries.

**Table 4:** Example of how the free-text answer was analysed following the Saldana coding (all quotes are Nurses´ quotes)

Examples from free text evaluation	Magnitude coding		Descriptive coding	In Vivo coding
	+	-		
The evaluation should take place after returning to practical work (about 4 weeks later)		Evaluation comes too early	Evaluation / practical reference	Evaluation some time after the course
Questions are too long and too many		Too many and extensive Items	Assessment sheet	“you lose the desire to answer”
I learned a lot and now practice with greater confidence	Learning gain		Global / Course	

#### Reporting

This is primarily a quantitative outcome evaluation study, with illustrative qualitative data. Our study provides a level 2 evaluation according to Kirkpatrick´s Model [28], as we evaluate the participants learning in relation to the transferred knowledge, skills and attitudes. In reporting the results, we followed the SQUIRE-EDU Guideline [29].

## Results

A total of 529 assessments were recorded (456 from nurses and 73 from physicians). Due to the small number of cases in other occupational groups, only the assessments of physicians and nurses were evaluated (see Table 5).

**Table 5:** Number of assessments by occupational group

Occupational group	Assessments
Nurses	456
Physicians	73
Psychologists	2
Physical therapists	16
Social workers	20
Health care chaplains	2
Misc	20

The competency fields with the highest and lowest learning gains were identical for nurses and physicians: the competency field "knowledge" showed the highest learning growth (45.43% physicians vs. 66.53% nurses), while "self-competence" (27.19% physicians vs. 48.84% nurses) showed the lowest learning growth. The competency field "skills" showed the second largest learning gain (41.88% p. vs. 60.20% n.), while "social competence" showed the third largest gain (31.44% p. vs. 54.58% n.). In summary, overall learning gains were higher for professional competencies (43.76% p. vs. 63.61% n.) than for personal competencies (29.48% p. vs. 51.95% n.) (see Table 6).

**Table 6:** Learning gain of nurses and physicians by competency field

Field of competence	**Learning gain [%]** **Nurses (*n* = 456)**	pre to post	**Learning gain [%]** **Physicians (*n* = 73)**	pre to post
Knowledge (Items 1–10)	66.53%	3.21 to.,74	45.43%	3.35 to 2.29
Skills (Items 11–20)	60.20%	2.84 to 1,73	41.88%	3.00 to 2.16
**Professional competence (Items 1–20)**	**63.61%**	3.03 to 1.74	**43.76%**	3.17 to 2.22
Social competence (Items 21–30)	54.58%	2.50 to 1,68	31.44%	2.71 to 2.17
Self-competence (Items 31–40)	48.84%	2.27 to 1.65	27.19%	2.44 to 2.05
**Personal competence (Items 21–40)**	**51.95%**	2.39 to 1.67	**29.49%**	2.58 to 2.11

The greatest learning gains were achieved by nurses in core competencies 2 “Improve the physical well-being of patients throughout their course of illness.” (65.60%) and 1 “Apply the core components of palliative care in the setting in which patients and families find themselves” (61.93%). The smallest learning gains were recorded in core competencies 3 “Meet the psychological needs of patients” (55.31%) and 10 “Practice self-awareness and engage in professional development” (53.68%). Overall, the greatest learning gain was achieved in the competence area "knowledge" (66.53%). "Self-competence" showed the smallest increase (48.84%).

Among physicians, core competencies 1 “Apply the core components of palliative care in the setting in which patients and families find themselves” (48.14%) and 8 “Practice comprehensive care coordination and interdisciplinary teamwork in all settings where palliative care is provided” (43.55%) achieved the greatest learning growth. Core competencies 3 “Meet the psychological needs of patients” (33.41%) and 10 “Practice self-awareness and engage in professional development” (25.85%) were identified as the core competencies with the lowest learning growth. Overall, the evaluation shows that each core competence and competence area experienced learning gains as a result of participating in training. On average, the learning gain was 30% less for physicians than for nurses.

Tables 6 & 7 show the learning gains for each field of competence and palliative care core competency by occupational group; see Additional file 1: Appendix 1 for a breakdown of learning gains for all items. The highest difference in mean pre-assessment ratings between the two occupational groups treated in this study is 0.21 (social competence 2.71 p. and 2.50 n.)

**Table 7:** Learning gain of nurses and physicians according to core competencies

**Core competencies: I am able to …**	**Learning gain [%]** **Nurses (n = 456)**	pre to post	**Learning gain [%]** **Physicians (n = 73)**	pre to post
… apply the core constituents of palliative care in the setting where patients and families are based (Items 1,11,21,31)	61.93%	2.68 to 1.64	48.14%	2.80 to 1.94
… enhance the physical comfort throughout patients´ disease trajectories (Items 2,12,22,32)	65.60%	2.66 to 1.57	39.49%	2.95 to 2.18
… meet patients´ psychological needs (Items 3,13,23,33)	55.31%	2.87 to 1.84	33.41%	3.20 to 2.46
… meet patients´ social needs (Items 4,14,24,34)	59.88%	2.89 to 1.76	41.08%	3.13 to 2.26
… meet patients´ spiritual needs (Items 5,15,25,35)	59.71%	2.85 to 1.74	35.16%	3.11 to 2.37
… respond to the needs of family carers in relation to short-, medium- and long-term patient care goals (Items 6,16,26,36)	59.98%	2.45 to 1.58	37.12%	2.56 to 1.98
… respond to challenges of clinical and ethical decision-making in palliative care (Items 7,17,27,37)	57.70%	2.62 to 1.69	39.22%	2.50 to 1.91
… practice comprehensive care coordination and interdisciplinary teamwork across all settings where palliative care is offered (Items 8,18,28,38)	58.29%	2.85 to 1.77	43.55%	2.85 to 2.04
… develop interpersonal and communication skills appropriate to palliative care (Items 9,19,29,39)	57.06%	2.47 to 1.63	36.59%	2.64 to 2.04
… practise self-awareness and undergo continuing professional development (Items 10,20,30,40)	53.68%	2.73 to 1.80	25.85%	3.01 to 2.49

### Answers from the free-text analysis

The analysis of the free-text answers shows a need for revision of the outcome-evaluation instrument in the categories of assessment, effort, comprehensibility and interprofessional collaboration (see Table 8). While an outcome evaluation is perceived as useful, this evaluation tool seems to be too long and partly difficult to understand: "After question ten, you lose the desire to answer" (Nurse – Participant Course No.2023).

**Table 8:** Participants´ quotes; processed and sorted according to Saldana

**Category**	**Quote**
**Assessment**	You should think about revising the form (Nurse's quote – P. Course No.2116)
	In my opinion the form is inaccurate and unnecessary (Nurse's quote – P. Course No.1875)
**Effort**	This form is more or less a burden (Nurse's quote – P. Course No.1695)
	This form is too much time-consuming (Nurse's quote – P. Course No.1695)
**Comprehensibility**	Questions are hard to understand (Nurse's quote – P. Course Lausitz)
	Questions are way too complicated (Nurse's quote – P. Course No.2116)
**Interprofessionality**	Not all (work areas) are to be found in the form (Nurse's quote – P. Course No.1655)
**Course / Content**	This was an hands-on, extensive and interesting course (Nurse's quote – P. Course No.1695)
**Learning gain**	I learned a lot and can be more confident now (Nurse's quote – P. Course No.2028)
	I got many information about palliative care offerings and in general the work on a palliative care-ward or outpatients. (Physician's quote – P. Course Sylt)
**Global**	On the whole this course was a great enrichment in a professional and personal way (Nurse's quote – P. Course No.2130)

In the category of practical relevance, a later assessment date is suggested, when the course content can be applied in practice “The evaluation should take place after returning into practical work (about 4 weeks later).” (Nurse – Participant Course No.1695).

The categories ‘course, content and learning gain’ include a global evaluation of the course.

The courses are perceived as enriching. An extension of the continuing education courses is suggested: "This course showed me how important it is to be open to lifelong learning, not to stop at knowledge already acquired and to reflect on one's points of view again and again, even if this dynamic process requires time, strength and courage" (Nurse – Participant Course No.1695). “The courses I did show me impressively how important an intensive palliative care is and that is far more than we do routinely in everyday oncology.” (Physician – Participant Course Sylt).

## Discussion

The main aim of this study is to present the ‘learning gains’ of nurses and physicians after participating in palliative care continuing education programmes in Germany. The specific outcome assessment demonstrated a learning gain for the participants from medical and nursing fields for all core competencies of palliative care, in all competence areas. Nevertheless, the adjusted physicians´ learning gains were 30% lower than nurses. Overall, the professional competencies (Knowledge and Skills) reached higher learning gains in comparison to the personal competencies (Social and Self competencies).

### Comparison with an IPE palliative care training in Ukraine

The outcome evaluation of a one-week interprofessional palliative care training in Ivano-Frankivsk, Ukraine provides comparable data [20]. Here, the present evaluation form was used with an English translation in a more heterogeneous group of test subjects (physicians, nurses, psychologists, social workers, and chaplains). The evaluation of the data also showed an increase in learning in all areas. Compared to the data of our cohort of physicians, the learning gain in personal competencies was higher in Paal et al. (social competence 44.55% Paal vs. 32.97% Marciniak, self-competence 44.58% Paal vs. 27.65% Marciniak), and professional competencies were also higher, but with a smaller difference (knowledge 54.94% Paal vs. 47.29% Marciniak, skills 51.42% Paal vs. 41.54% Marciniak). There was also an increase in learning levels for all core competencies (average 48.87% Paal vs. 37.36% Marciniak). When comparing the data collected, the heterogeneity of the Paal et al. cohort must be considered. It is possible that a comparison of the physicians´ data would yield a smaller difference potentially due to ceiling effects. In addition, the course offered lectures as well as self-reflection exercises, topic-based discussions, and profession-specific workshops. By using this evaluation internationally, continuing education courses could be compared and further developed through a regular exchange.

### Palliative care education in Germany

Overall, the learning gain is greater for the nurses in all sub-areas of the evaluation. In Germany, nurses are trained in applied universities. There are no systematically obtained data that provides information on how palliative care is taught in German nursing schools. However, this circumstance should not affect the comparability; because of the previously explained CSA Gain formula that considers pre-education levels.

Overall a high degree of transparency and comparability should be targeted when talking about enhancements in continuing education/content of teaching in palliative care. As a future concept, it may be useful to engage the implementation of interprofessional continuing education programmes. If nurses and physicians attend the same courses this could provide an exchange of experience, and a better understanding of the other occupational groups’ roles that may improve teamwork [30].

A strength of this study is the retrospective “learning gain” analytical technique. In the Stanford Faculty Development Centre's End-of-Life Care Program, learning gains were calculated with and without retrospective assessment. The traditional pre-test ratings in this study were higher than the retrospective ones. Both approaches of evaluation can provide valid data, nevertheless, the retrospective way of employing response shift analysis is likely to present more sensitive and valid measures in showing the effect of learning gains [31]. The distribution of the learning gain among the core competencies of palliative care seems balanced. Assuming that the core competencies take all relevant teaching content into account, this distribution could indicate that the evaluated courses reflect the relevant teaching content of palliative care.

### Theoretical versus hands-on courses

The consideration of the competence areas shows that the learning gain in the area of professional competence, especially knowledge, is significantly greater in both professions than in the area of personal competence, especially self-competence. The learning gain was calculated using a specific formula and, in this way, different levels of prior learning could be calculated and a comparable learning gain could be presented. The question arises whether postgraduate education in the form of theoretical courses is sufficiently suitable for teaching aspects relevant to palliative care, such as social and self-competence. To investigate this question, a follow-up project will compare the learning gain in the context of case seminars with that of an internship in a palliative care ward. In assessing the results, it should also be noted that the underlying assessment measure was a comparative retrospective self-assessment. No examinations were conducted at the end of the course. Although it was suggested that a post-test self-assessment with response shift data is equivalent to a pre-post evaluation, the risk of response tendencies or response bias must be considered, just as with the pre-post approach [25]. Complete anonymization of the assessment should minimise such effects, but cannot be ruled out.

It should also be considered whether an improvement in learning gains in the area of skills and personal competence could be achieved by optimising the teaching components within the training programmes, e.g. by strengthening interprofessional education and clinical reasoning in the course—analogous to the development of palliative medicine teaching in the context of undergraduate medical training [32, 33] and undergraduate nursing [34].

### Optimising evaluation

**Fig. 1:**
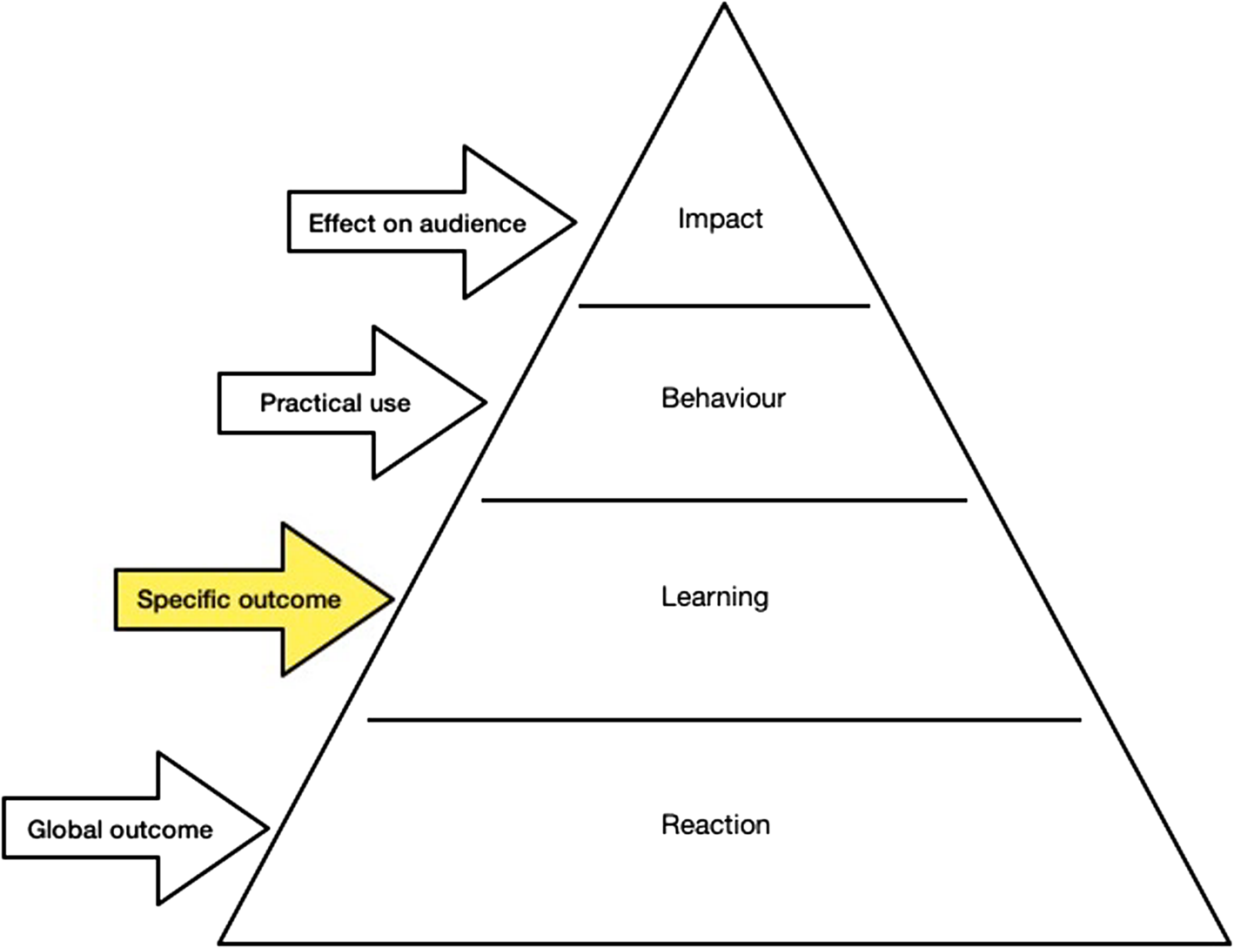
Classification in the four-stage evaluation model according to Kirkpatrick

In parallel, a revision of the evaluation approach is necessary to ensure acceptance by participants in the future. The sustainability of the effects of the training as well as the implementation of the competencies in clinical practice, in the respective disciplines and professional groups, must be verified. Following Kirkpatrick's evaluation model, an additional evaluation point to capture practical implementation (level 3 behaviour) and a survey of the patient perspective (level 4 impact) should be discussed (see Fig. 1).

In 2022, Noguera presented a study, specifically addressing Kirkpatrick´s levels [35] where changes in behaviour and the impact of the Palliative Care courses on students were discussed/evaluated by teachers. Via this paper, we get a rare evaluation of palliative education from the teachers’ side, with suggestions as to how future evaluation could be conducted.

### Limitations

No personal data were collected, so no statement can be made about the age and professional experience of the participants, except for their minimum degrees. All physicians were licensed or owned the German ‘Approbation’ and all nurses were qualified via a state examination. With regard to the nurses´ qualifications, it is important to remember that the German state examination is not the same as the advanced practice nurse or the clinical nurse specialist degree. Furthermore, the response rate cannot be calculated because we have no information on whether all participants in a course took part in the assessment. However, since the assessments received per course roughly correspond to the DGP recommendations on maximum group size, a high response rate (at least 80%) can be assumed.

For the future, it would be conceivable to carry out further / new data collection. In this context, it would be important to ask for further details (i.e. career stages, experience, training, demographics) of the probands.

The number of nurses in our study is significantly higher than that of physicians (456 vs 73). However, examining the approximate distribution of occupational groups in the health care system (ca 80% nurses vs 20% physicians), our dataset reflects this.

The wording of the learning objectives was taken verbatim from KoMPaC to ensure that the learning objectives are reflected in the content. This perhaps leads to an open interpretation of the learning objective formulation for some elements.

All of our data is extracted from subjective self-assessments. Although the results have a high potential to illustrate learning gains and strengths and weaknesses of the actual continuing education programmes, we can't use these as absolute percentages of learning gain.

When interpreting the collected data, a certain selection bias must be considered. Our assessment took place in voluntary courses; in Germany, continuing education in palliative care is a very specialised education. It can be assumed that most of the participants hoped for knowledge gain and good education. Based on this assumption the considered bias is noted and we have to accept this bias because of the existing sample population.

## Conclusions

The distribution of learning gains among the individual core competencies and fields of competence gives the impression in both professional groups that the areas of personal competencies and skills are underdeveloped, although they play an important role in everyday palliative care. This phenomenon is already known from palliative care training in undergraduate education [36]. Currently, there is a positive development towards a stronger consideration of psychomotor and affective learning goals, even if these are still insufficiently considered in examinations [33].

To optimise learning gains in the areas of skills and personal competence, an adaptation of didactic concepts should be discussed and specific training for teachers considered. According to Noguera et al., a well-trained teacher is needed to transport information [35]. For example, post-graduate training could be proportionately interprofessional to promote communication between the professions; such projects exist, for example, in the area of undergraduate education and training [37]. Furthermore, the development of Entrustable Professional Activities (EPA) seems to make sense to strengthen the roles of the individual, for example, as a communicator, team member or professional [38].

## Supplementary Information

**Additional file 1.** 

## Data Availability

The dataset during and/or analysed during the current study is available from the corresponding author on reasonable request.
