# Supplementary material for: The outcomes of postgraduate palliative care education and training: assessment and comparison of nurses and physicians
Source: BMC Palliat Care. 2023 Jul 13;22:94. doi: 10.1186/s12904-023-01217-1 (PMC10339497; doi:10.1186/s12904-023-01217-1)
Supplement: Supplementary file 1 — Additional file 1. [file 12904_2023_1217_MOESM1_ESM.docx]

Appendix 1

| Item | Learning gain [%]  Nurses (n=456) |  | Learning gain [%]  Physicians (n=73) |  |
| --- | --- | --- | --- | --- |
| 1. I can explain the meaning of life-limiting ilness to the person concerned | 63.05% | 2.82 to 1.66 | 53.14% | 2.51 to 1.72 |
| 1. I can explain the Total Pain concept in detail | 68.42% | 3.86 to 1.87 | 41.14% | 3.96 to 2.88 |
| 1. I can name essential characteristics of fear, demoralization and depression | 58.97% | 2.95 to 1.77 | 36.04% | 3.25 to 2.45 |
| 1. I can explain the main social needs of those affected in the context of their impact of palliative care | 69.62% | 3.19 to 1.65 | 62.09% | 3.35 to 1.90 |
| 1. I can explain the meaning of rituals at the end of life | 72.34% | 3.10 to 1.57 | 47.83% | 3.48 to 2.29 |
| 1. I can name risk factors of family caregivers | 65.74% | 2.89 to 1.63 | 52.59% | 3.29 to 2.10 |
| 1. I can explain the relevant legal and ethical principles of care for seriously ill and dying patients | 61.02% | 3.72 to 2.05 | 46.05% | 3.79 to 2.51 |
| 1. I can name all outpatient and inpatient palliative care structures | 71.28% | 3.38 to 1.66 | 55.40% | 3.54 to 2.15 |
| 1. I can substantiate the importance of interdisciplinary and inter-professional cooperation | 67.09% | 3.02 to 1.65 | 58.05% | 2.59 to 1.68 |
| 1. I can name all the relevant risk factors of burnout development process | 58.01% | 3.20 to 1.92 | 20.55% | 3.77 to 3.19 |
| 1. I adopt the palliative care approach as early as possible in the disease process | 73.01% | 3.20 to 1.58 | 52.04% | 3.46 to 2.19 |
| 1. I delevop individual strategies to actively support the patient´s wellbeing and quality of life to maintain the patient´s dignity | 72.98% | 2.36 to 1.37 | 56.75% | 2.74 to 1.76 |
| 1. I recognize health care supply related resources and risk related to individual family structures | 60.58% | 2.99 to 1.77 | 49.27% | 3.18 to 2.10 |
| 1. I follow explicitly the fundamentals of various consulting and communication methods | 59.64% | 3.26 to 1.88 | 42.66% | 3.56 to 2.48 |
| 1. I identify possible questions of meaning and conscience related to imminent death | 60.96% | 3.17 to 1.83 | 43.61% | 3.37 to 2.33 |
| 1. I involve members and associates in discussions and decision-making | 64.61% | 2.32 to 1.45 | 38.83% | 1.86 to 1.51 |
| 1. I make and implement decisions in the patient´s and relatives´interest | 54.36% | 2.39 to 1.62 | 32.33% | 2.09 to 1.74 |
| 1. I coordinate necessary support and care options both within and beyond the team | 44.65% | 3.32 to 2.27 | 35.17% | 3.46 to 2.60 |
| 1. I definitive use context- and person appropriate vocabulary | 53.55% | 2.40 to 1.63 | 35.58% | 2.70 to 2.10 |
| 1. I routinely implement self-care strategies | 52.65% | 2.96 to 1.91 | 29.15% | 3.53 to 2.79 |
| 1. I always consider the patient and their family members as experts in their own lives | 54.92% | 2.28 to 1.57 | 40.38% | 2.34 to 1.83 |
| 1. I always perceive and acknowledge the patient´s individual symptom perception and suffering experience | 63.32% | 2.19 to 1.43 | 47.30% | 2.47 to 1.77 |
| 1. I can evaluate patient´s psychological symptoms in a structured way | 51.33% | 2.94 to 1.92 | 26.67% | 3.61 to 2.92 |
| 1. I can identify my personal challenges and limitations in the presence of patients and their relatives | 53.70% | 2.63 to 1.74 | 27.14% | 2.83 to 2.33 |
| 1. I always support the bereavement and loss process of those affected | 55.81% | 2.41 to 1.63 | 23.05% | 2.90 to 2.46 |
| 1. I provide professional security and confirmation to caregivers and relatives | 55.59% | 2.37 to 1.60 | 28.25% | 2.75 to 2.26 |
| 1. I always allow family members and relatives to be involved in the decision-making process | 53.08% | 2.33 to 1.61 | 32.57% | 2.17 to 1.79 |
| 1. In the palliative care setting, I generally promote multi-professional teamwork | 59.70% | 2.52 to 1.60 | 47.13% | 2.23 to 1.66 |
| 1. In my professional role, I always respond adequately tot he emotional reactions of interviewees | 52.48% | 2.36 to 1.63 | 28.63% | 2.66 to 2.18 |
| 1. I routinely build up an appreciative error culture | 43.40% | 3.01 to 2.11 | 28.54% | 3.10 to 2.49 |
| 1. I always use my own resources cautiously and team-oriented | 45.45% | 2.42 to 1.75 | 48.61% | 2.90 to 1.99 |
| 1. I essentially respect my own and others´ limits | 48.49% | 2.23 to 1.61 | 19.22% | 2.60 to 2.30 |
| 1. I implement a high degree of self-reflection in all areas of my professional activity | 43.72% | 2.62 to 1.89 | 20.40% | 2.75 to 2.39 |
| 1. I differentiate professionally my own values from those of the patient | 47.21% | 2.46 to 1.76 | 26.05% | 2.79 to 2.32 |
| 1. I regularly reflect on my own meaning of life | 43.64% | 2.70 to 1.95 | 17.91% | 2.68 to 2.38 |
| 1. I respect professionally the existing social system (family and friends) | 45.98% | 2.23 to 1.65 | 21.99% | 2.35 to 2.05 |
| 1. I always respect the autonomous decisions of the patient and their relatives and friends | 53.72% | 2.04 to 1.47 | 37.33% | 1.96 to 1.62 |
| 1. I always show the willingness to develop and seek conflict situations | 53.45% | 2.16 to 1.55 | 34.60% | 2.16 to 1.77 |
| 1. I always give praise and criticism within and beyond the team with tact | 43.12% | 2.09 to 1.60 | 24.48% | 2.61 to 2.21 |
| 1. I apprehend that up-to-date knowledge is not static and needs to be continuously developed and expanded in the process of lifelong learning | 63.41% | 1.78 to 1.27 | 25.87% | 1.65 to 1.49 |
